# APP Is Cleaved by Bace1 in Pre-Synaptic Vesicles and Establishes a Pre-Synaptic Interactome, *via* Its Intracellular Domain, with Molecular Complexes that Regulate Pre-Synaptic Vesicles Functions

**DOI:** 10.1371/journal.pone.0108576

**Published:** 2014-09-23

**Authors:** Dolores Del Prete, Franco Lombino, Xinran Liu, Luciano D'Adamio

**Affiliations:** 1 Department of Microbiology & Immunology, Albert Einstein College of Medicine, Bronx, New York, United States of America; 2 Department of Cell Biology, Yale University School of Medicine, New Haven, Connecticut, United States of America; Nathan Kline Institute and New York University Langone Medical Center, United States of America

## Abstract

Amyloid Precursor Protein (APP) is a type I membrane protein that undergoes extensive processing by secretases, including BACE1. Although mutations in *APP* and genes that regulate processing of APP, such as *PSENs* and *BRI2/ITM2B*, cause dementias, the normal function of APP in synaptic transmission, synaptic plasticity and memory formation is poorly understood. To grasp the biochemical mechanisms underlying the function of APP in the central nervous system, it is important to first define the sub-cellular localization of APP in synapses and the synaptic interactome of APP. Using biochemical and electron microscopy approaches, we have found that APP is localized in pre-synaptic vesicles, where it is processed by Bace1. By means of a proteomic approach, we have characterized the synaptic interactome of the APP intracellular domain. We focused on this region of APP because *in vivo* data underline the central funtional and pathological role of the intracellular domain of APP. Consistent with the expression of APP in pre-synaptic vesicles, the synaptic APP intracellular domain interactome is predominantly constituted by pre-synaptic, rather than post-synaptic, proteins. This pre-synaptic interactome of the APP intracellular domain includes proteins expressed on pre-synaptic vesicles such as the vesicular SNARE Vamp2/Vamp1 and the Ca^2+^ sensors Synaptotagmin-1/Synaptotagmin-2, and non-vesicular pre-synaptic proteins that regulate exocytosis, endocytosis and recycling of pre-synaptic vesicles, such as target-membrane-SNAREs (Syntaxin-1b, Syntaxin-1a, Snap25 and Snap47), Munc-18, Nsf, α/β/γ-Snaps and complexin. These data are consistent with a functional role for APP, *via* its carboxyl-terminal domain, in exocytosis, endocytosis and/or recycling of pre-synaptic vesicles.

## Introduction

Alzheimer's disease (AD) is the most common cause of dementia in the world. Mutations in *APP* were linked to familial AD ∼20 years ago [1]; yet, the molecular mechanisms underlying APP physiological function remain elusive due to the complex APP metabolism and the presence of the functionally redundant genes *APP like protein-1* and *-2* (*APLP1/ALPL2*) [2]–[6]. APP is cleaved by β-secretase (BACE1) into sAPPβ and β-CTF. Cleavage of β-CTF by the γ-secretase yields Aβ and AID/AICD peptides. Alternatively, α-secretase clips APP into sAPPα and α-CTF. α-CTF is cut by γ-secretase into P3 and AID. In addition to *APP*, mutations in genes that regulate APP processing, such as *PSENs* and *BRI2/ITM2B*, cause dementias [1], [7]–[12]. This evidence emphasizes the pathogenic role of APP processing in familial human dementias.

APP is involved in synapse formation, dendritic spine formation, synaptic transmission, neurites outgrowth, learning and memory, motility and development [13]. Though all APP metabolites have biological functions [14]–[20], phenomenological observations underline the key physiological and pathological role of the APP intracellular domain [21]–[26]. Mutation of single APP intracellular residues can have dramatically opposite effects. For example, mice carrying the Y^682^G (using the APP-695 isoform numbering) mutation in the intracellular domain, present functional deficits similar to that of *APP* KO mice, including cognitive and neuromuscular junctions deficits [23], [25]. On the opposite, the T^668^A mutation in the intracellular domain prevents the development of synaptic and memory deficits of FDD_KI_ mice, a model of the AD-like Familial Danish dementia [21], which is due to mutations of *BRI2/ITM2B*, and inhibitor of APP processing by BACE1 [7]–[9]. The functional and pathological relevance of the APP intracellular region is also stressed by evidence showing that β-CTF impairs spatial learning and synaptic plasticity in mice [27] and plays a pathogenic role in synaptic and memory deficits of FDD_KI_ mice [28]–[32].

In spite of the important pathogenic role of APP, the molecular and biochemical mechanisms underlying the function of APP and, more specifically, its intracellular region remain unclear. Defining the sub-cellular localization of APP and APP-derived metabolites (especially those containing the APP intracellular domain, i.e. full-length APP, β-CTF, α-CTF and AID/AICD) in synapses, and characterizing the synaptic interactome of the APP intracellular domain would represent an important step-forward in understanding the function of APP in synaptic transmission, synaptic plasticity and memory formation. Here, we have utilized biochemical and electron microscopy approaches to decipher the precise localization of APP and APP-COOH-terminal fragments (APP-CTF) in synapses. To gain information concerning the sub-cellular compartments where APP is processed, we have determined whether APP and the APP-cleaving enzymes Bace1 and γ-secretase are co-localized in similar synaptic fractions. Our results indicate that both APP and Bace1 are located in pre-synaptic vesicles and that Bace1 cleaves APP in pre-synaptic vesicles. To determine the synaptic interactome of the APP intracellular domain, we have used an unbiased proteomic approach. Consistent with the preferential pre-synaptic localization of APP, and more specifically the localization to pre-synaptic vesicles, the proteomic experiments show that the synaptic interactome of the APP intracellular domain is composed mainly of proteins expressed on or associated with pre-synaptic vesicles, as well as the active zone. Thus, we will refer to it as the AID-pre-synaptic interactome (AID-pre-sy-iome). Remarkably, the vast majority of the proteins composing the AID-pre-sy-iome play a pivotal role in exocytosis, endocytosis and recycling of pre-synaptic vesicles.

## Results

### Sub-cellular fractionation of mouse brains and characterization of fractions

**Figure 1:**
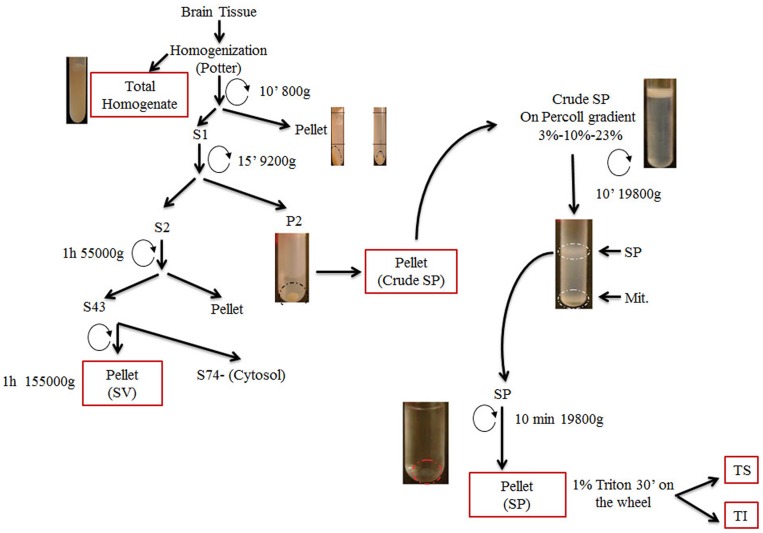
Schematic illustration of the fractionation protocol. Mouse brain tissues were homogenized in homogenization buffer (HB) and centrifuged. The S1 supernatant fraction was collected and further centrifuged obtaining supernatant (S2) and pellet (P2) fractions. The P2 fraction was diluted in HB and directly applied to a discontinuous Percoll gradient to obtain the synaptosome fraction (SP) which was then lysed using 1% TritonX-100 to yield soluble (TS) and insoluble fractions (TI). The S2 fraction was ultra-centrifuged in two different steps to obtain two pellet fractions (P43 and SV) and supernatant (S74).

**Figure 2:**
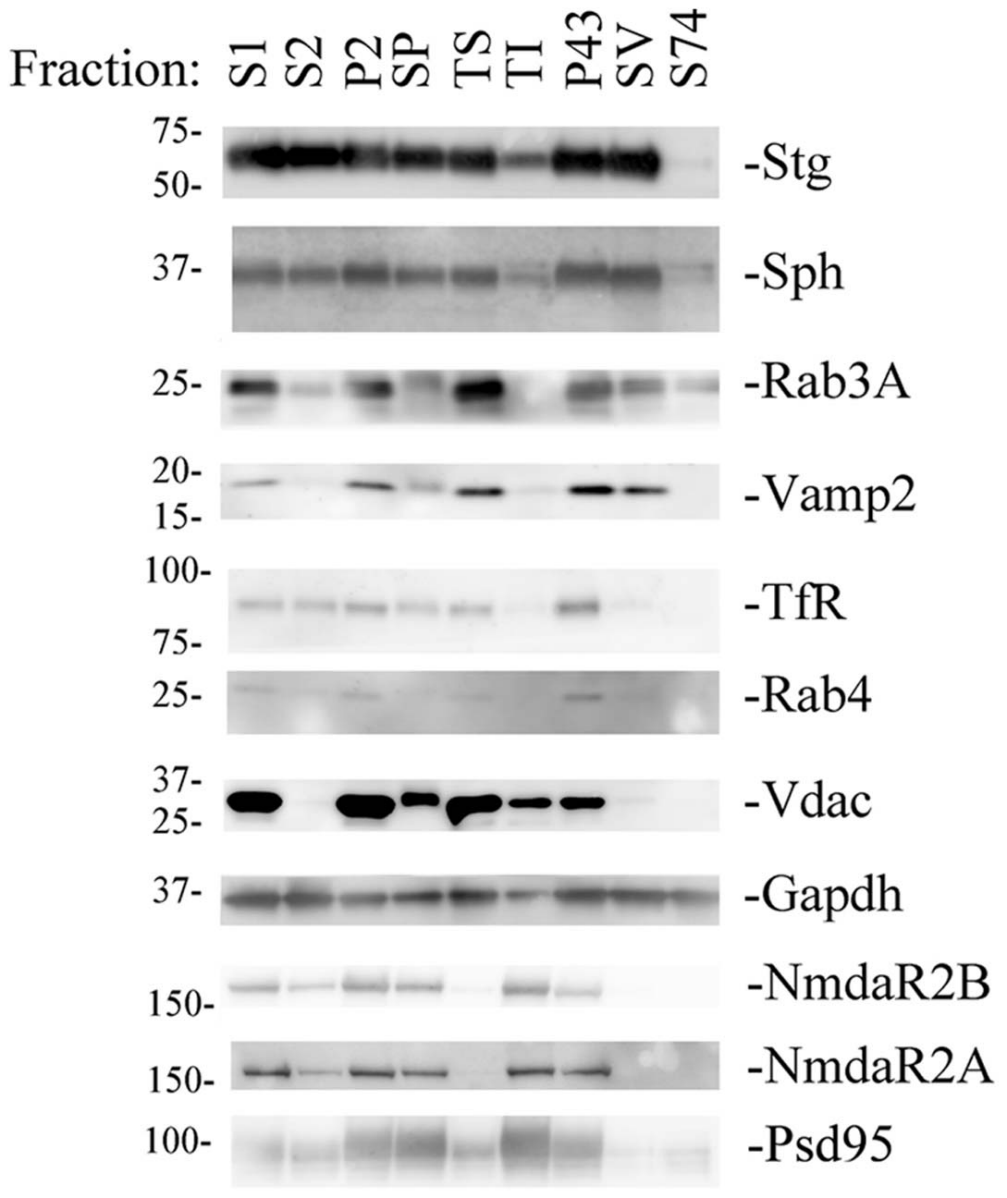
Analysis of the efficacy of the fractionation method. The same amount of protein from each fraction was subjected to SDS-PAGE, followed by immuno-blotting for various marker proteins. Antibodies for Stg, Sph, Rab3A and Vamp2 were used as marker for pre-synaptic vesicles; the antibody for Vdac was used as markers for mitochondria; the antibodies for Rab4 and TfR were used as markers for endosomes: the antibodies for NmdaR2A, NmdaR2B and PSD95 were used as marker post-synaptic membrane. Anti-Gapdh was used to normalize the loading.

To obtain fractions enriched in pre-synaptic vesicles, we adopted a fractionation protocol for brain homogenates that is schematically shown in Fig. 1. The first centrifugation step at 800 gravities (g) removes the nuclei and cell debris from the other organels and soluble proteins that remain in the supernatant fraction S1. Centrifugation of the S1 fraction at 9200 g separates the synaptosomes (SP), along with contaminating plasma membranes, myelin and mitochondria (P2 fraction), from small synaptic vesicles, endoplasmic reticulum and soluble material that are left in the supernatant fraction S2. The P2 fraction is dissolved in homogenization buffer and applied to a discontinuous Percoll gradient comprising layers of 3% (vol/vol), 10% (vol/vol), and 23% (vol/vol) Percoll. The gradient is then centrifuged at 19800 g for 10 min. Three major fractions are collected from the interfaces between the Percoll layers: Fraction 1 that contains myelin, membranes and membrane vesicles; Fraction 2, which is enriched in SP and membrane vesicles; Fraction 3, which contains extra-synaptosomal mitochondria. SP is washed and then lysed using 1% TritonX-100 to yield triton-soluble (TS) and triton-insoluble fractions (TI). The S2 fraction is centrifuged at 55000 g to obtain S43-supernatant -containing small synaptic vesicles and soluble proteins- and P43 –enriched in microsomes and lysosomes. S43 is further centrifuged at 160000 g to obtain synaptic vesicles (SV) in the pellet and soluble proteins in the supernatant (S74). Successful separation of the various sub-cellular components, as well as the degree of contamination by other organelles and particles, is monitored by immunoblotting the fractions obtained during the purification for known marker proteins. The SV fraction was highly positive for Synaptophysin (Sph), Synaptobrevin (Vamp2), Synaptotagmin (Stg) and Rab3A –which are known constituents of pre-synaptic vesicles. Proteins that are mainly localized in the post-synaptic density (Psd95, NmdaR2A, Nmdar2B) were enriched in the triton insoluble (TI) SP fractions, as expected. The SV fraction is largely devoided of mitochondria (as determined by Western blot for the mitochondrial protein Vdac) and endosomes (as determined by the absence of the endosomal proteins Rab4 and transferrin receptor, Tfr) (Fig. 2).

### APP, Bace1 and APP-metabolites derived by Bace1 processing of APP -β ˜–CTF and sAPPβ- are detected in synaptic vesicle fractions

**Figure 3:**
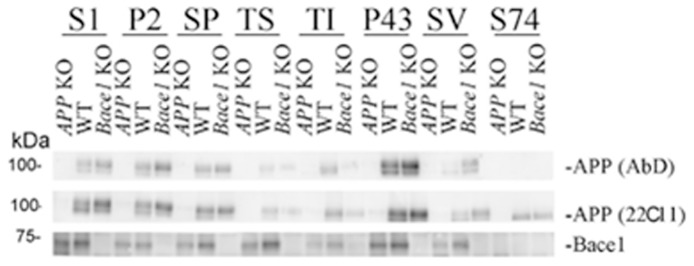
APP and Bace1 are found in SV fractions. Western blot analysis was used to determine the levels of APP and Bace1 in the different brain fractions. *APP* KO and *Bace1* KO mice fractions were used as controls for the specificity of the APP and Bace1 signals observed in the corresponding fractions isolated from WT brains.

We performed sub-cellular fractionation of brains from *APP* KO, Wild-type (WT) and *Bace1* KO mice. We first estimated the levels and distribution of full-length APP and Bace1 in the fractions collected. Bace1 and full-length APP, detected by both an antibody against the C-terminal (AbD) and N-terminal (22C11) region of APP, were widely distributed in fractions that contain cellular membranes, including the SV fraction. As expected, no Bace1 and full-length APP were detected in the fraction containing soluble proteins (S74). However, S74 contains APP-derived metabolites that are recognized by 22C11 but not AbD (Fig. 3). In all probability, these metabolites represent the soluble-APP ectodomains sAPPα and sAPPβ, which are shed by cleavage of full-length APP by α- and β-secretase (Bace1), respectively.

**Figure 4:**
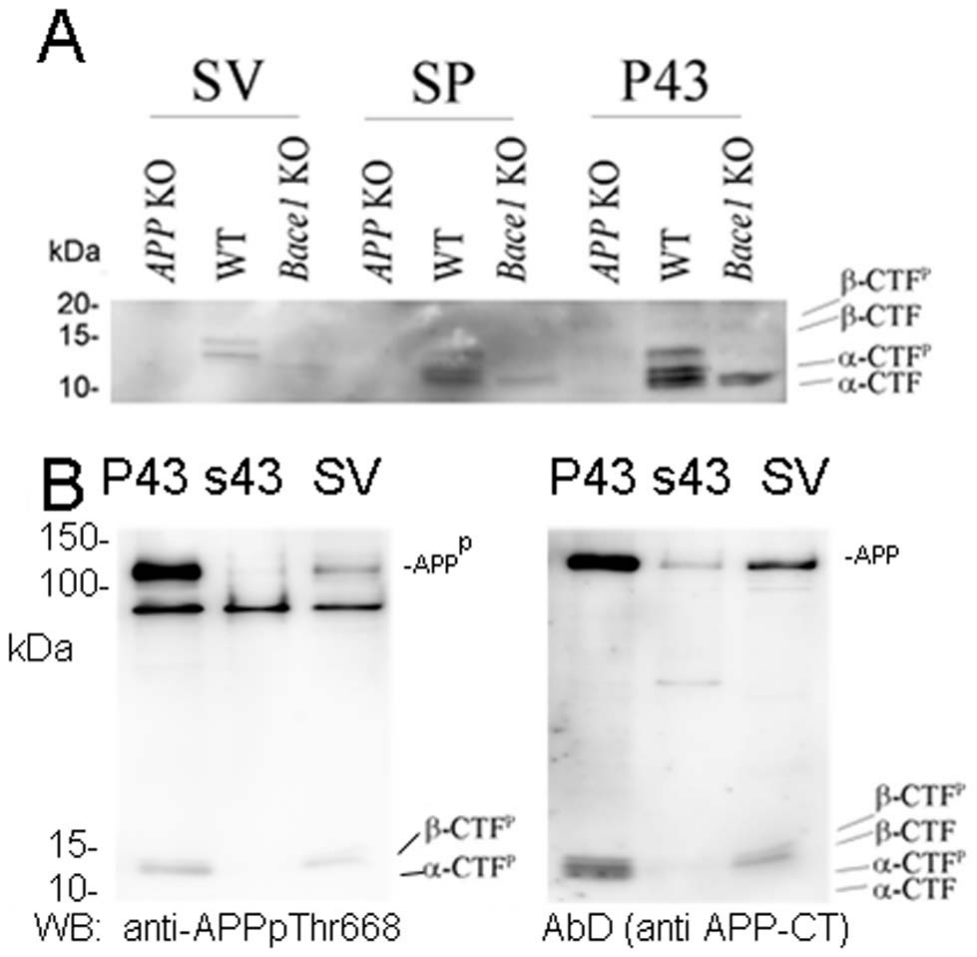
The APP metabolite β-CTF, but not α-CTF, is found in SV fractions. A) Analysis of APP-CTFs in membranous fractions of WT, *APP* KO and *Bace1* KO mouse brains. Only β-CTF fragments are detected in the SV fraction of WT mice. Traces of α-CTF are seen in the *Bace*1 KO SV fraction. Since we did not detect APP-CTF in other SV preparations from *Bace*1 KO mice (see Fig. 5 for example), we conclude that this signal probably indicates a minute contamination of this SV preparation with other membranous fractions. B) Western blot analysis of P43, the soluble fraction s43 and SV with an anti-APPpThr668 antibody confirms the presence of APP and CTFs phosphorylated on Thr^688^. Of note, the membrane bound APP and APP-CTFs are absent, as expected, in the soluble fraction. Again, while α-CTF, α-CTF phosphorylated on Thr^668^ (α-CTF^p^), β-CTF and β-CTF phosphorylated on Thr^668^ (β-CTF^p^) are all present in the P43 fraction, only β-CTF and β-CTF^p^ are detected in the SV fraction.

Next, we investigated the presence of APP carboxyl-terminal fragments (APP-CTFs) in SP, SV and p43 fractions. To obtain a better parting of APP-CTFs, samples were separated on a 16.5% Tris-Tricine PAGE. As shown in Fig. 4A, in the p43 fraction of WT mice we detected several APP-CTF species. All those species are specific since they are not seen in the *APP* KO sample. The higher species are absent in the *Bace1* KO sample, indicating that they derive from Bace1-mediated processing of APP. We will refer to these forms as β-CTFs. The CTF species that are still present in the p43 fraction isolated from *Bace1* KO brains are probably derived from α-secretase processing of APP and will therefore be referred to as α-CTFs. The presence of multiple β-CTF and α-CTFs species reflects phosphorylation of β-CTF and α-CTFs, probably at Thr^668^ [22], [33]. These conlusions were confirmed performing a WB analysis with an antibody specific for APP and APP-CTFs phosphorylated on Thr^668^ (Fig 4B). All APP-CTF species were present in the SP fraction of WT mice, while only α-CTFs peptides were detected in the corresponding *Bace1* KO sample. Interestingly, the SV fraction of WT mice contained only β-CTF species and not α-CTF. The absence of these APP-CTF fragments in the SV fraction of *APP* KO and *Bace1* KO mice confirms that the APP-CTFs detected in the WT SV fraction are indeed the product of Bace1-processing of APP (Fig. 4).

**Figure 5:**
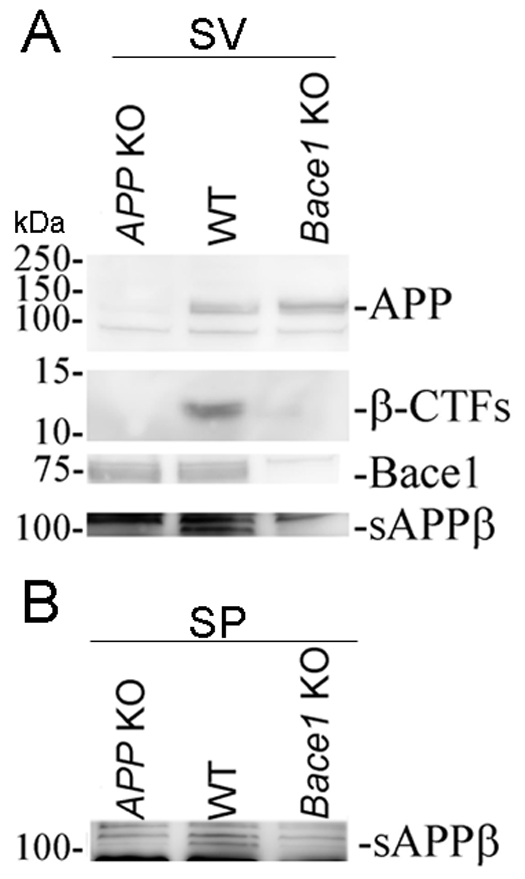
Evidence that Bace1 cleaves APP in pre-synaptic vesicles. **A**) Equal amounts of proteins from SV fractions of WT, *APP* KO and *Bace1* KO mice were subjected to SDS-PAGE, followed by immuno-blotting for APP (using AbD), Bace1 and sAPPβ. sAPPβ was detected in the SV fraction of WT mice supporting the hypothesis that Bace1 cleaves APP in pre-synaptic vesicles. B) sAPPβ was also detected in the SP fraction of WT mice.

Since Bace1 has an optimum activity at pH 4.5, Bace1 is primarily active in acidic compartments, such as late endosomes and lysosomes [34]. Notably, the lumen of pre-synaptic vesicles is acidic (pH 5.6), which is compatible with Bace1 activity. Thus, the pH of pre-synaptic vesicles, together with the presence of Bace1, full-length APP and β-CTF species in SV fractions suggests that Bace1 cleaves APP in pre-synaptic vesicles. Alternatively, the β-CTF species present in pre-synaptic vesicles may be produced in other sub-cellular compartments and accumulate in pre-synaptic vesicles subsequently. We reasoned that, if Bace1 cleaves APP in pre-synaptic vesicles, then sAPPβ should be found in the lumen of pre-synaptic vesicles. If instead the β-CTF species present in pre-synaptic vesicles are formed in other organelles, then sAPPβ will probably not be present in the lumen of pre-synaptic vesicles. To test for this, we prepared again SV fractions from WT, *APP* KO and *Bace1* KO mice. Western blot analysis confirmed that Bace1, full length APP and β-CTF, but not α-CTF, species are present in the SV fraction of WT mice (Fig. 5A). Again, no APP-CTFs were seen in *APP* KO and *Bace1* KO SV samples (Fig. 5)A. Next, we performed an immunoblot analysis on SV fractions using an antibody that specifically recognizes sAPPβ. This antibody detects non-specific signals at around 110 kDa (signals that are also present in *APP* KO and *Bace1* KO SV fractions) but also a specific band of size compatible with sAPPβ that is only detected in the SV fraction of WT mice (Fig. 5A). Western blot analysis of SP fractions also showed the presence of sAPPβ in the SPs (Fig. 5B), suggesting that these sAPPβ species are could derive from pre-synaptic vesicles present in the SP fraction (for example those docked to the active zone.

**Figure 6:**
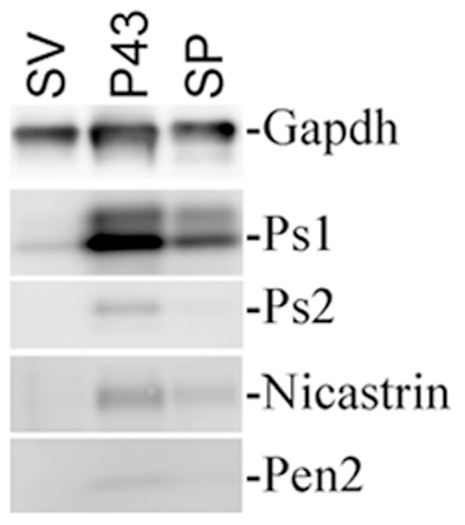
Minimal levels of γ-secretase components are found in SV fractions. Analysis of **γ**-secretase subunits distribution in brain fractions isolated from WT mice. SV, P43 and SP fractions were prepared using the protocol described in Fig. 1 and analyzed for the presence of the **γ**-secretase components Ps-1, Ps-2, Nct and Pen2 using Western blots. While all four γ-secretase components were readily detected in the P43 and, albeit at lower levels, SP fractions, only Ps-1 was detected, at very minimal level, in the SV fraction. Gapdh was used to normalize the loading.

The evidence that Bace1 cleaves APP in SV fraction and that Aβ production is increased by synaptic activity [35], prompted us to test whether γ-secretase, i.e. the protease that generates Aβ from its precursor β-CTF, is present in our SV preparations. γ-secretase is a membranous complex consisting of at least four proteins: Presenilin-1 or -2 (Ps-1 and Ps-2), Nicastrin (Nct), anterior pharynx defective-1 (Aph-1) and presenilin enhancer-2 (Pen-2). While the γ-secretase components Nct, Ps-1, Ps-2 and Pen-2 are readily detectable in the SP and P43 membrane-fractions, only small amounts of Ps-1 were detected in the SV fraction (Fig. 6).

Overall, the acidic pH of pre-synaptic vesicles, the presence of full-length APP, Bace1, β-CTF and sAPPβ in SV fractions supports the hypothesis that Bace1 cleaves APP in pre-synaptic-vesicles. The evidence that only traces of some γ-secretase components are seen in our SV fractions would suggest that β-CTF species are not cleaved into Aβ and AID/AICD in pre-synaptic-vesicles. However, specially considering that others have instead reported the presence of some γ-secretase components in biochemical preparations of SV fractions from rat brains [36], further work is required to conclusively determine whether pre-synaptic-vesicles contain sufficient levels of γ-secretase to turn over β-CTF *in situ*.

### Immuno-EM localization of APP in hippocampal pre-synaptic vesicles

**Figure 7:**
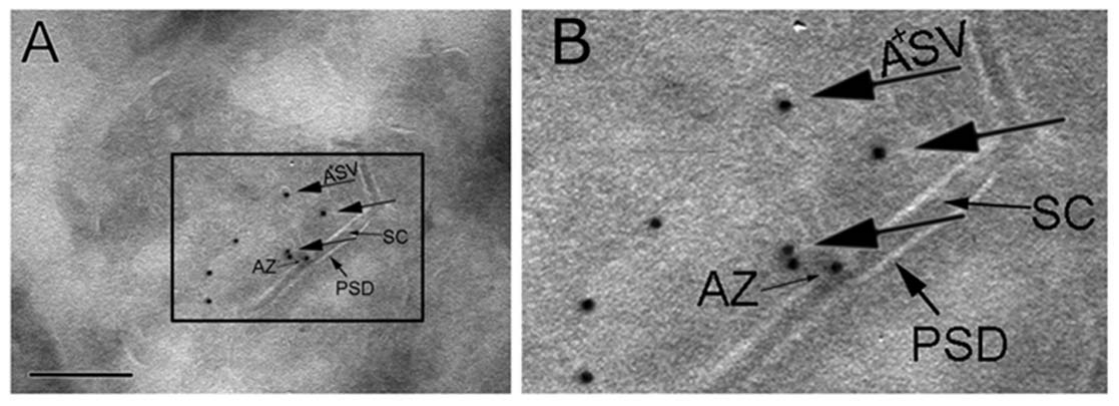
APP localizes in pre-synaptic vesicles. **A**) and **B**) Immuno-EM with the anti-APP-C-terminal antibody Y188 in hippocampus of wild type mouse brain shows APP in SV. We have used Y188 because this anti-APP antibody has demonstrated specificity for APP in Immuno-fluorescence experiments. The experiment was performed using cryosectioning, and immunogold labeling technique. Synapses were identified by morphology, i.e. SV, clefts (SC), the active zone (AZ) and the post-synaptic density (PSD). Larger arrows pointing gold particles (10 nm) indicate distribution of APP predominately on SV (A+SV). Scale bar in A is 250 nm. B) Enlarged version of a part of A.

Assigning the sub-cellular localization of endogenous proteins solely based on biochemical fractionations experiments can be risky, since biochemical approaches can seldom achieve 100% purity. In this context, our biochemical evidence that Bace1 is contained in pre-synaptic-vesicles is consistent with recent findings that have conclusively localized Bace1 in pre-synaptic-vesicles by immune-Electron microscopy (I-EM) [37]. Thus, we decided to use I-EM to localize APP and APP-metabolites in CA1 synapse. We utilized the Y188 antibody, which was raised against the C-terminal sequences of APP and therefore detects full length APP and APP metabolites containing the intracellular domain -such as β-CTF and α-CTF. In theory also AID/AICD could be detected by Y188. However, given the minimal steady-state levels of this APP metabolite, we tend to exclude that Y188 will detect AID/AICD in I-EM. We selected the Y188 antibody because in a screening of several anti-APP antibodies that have been routinely used to determine APP expression and localization using immune-histo-chemistry and I-EM, Y188 was the only one that showed strong specificity for APP in brain and primary neuronal cultures [38]. As shown in Fig. 7, the positive immunoreactive signals are predominately associated with pre-syanptic vesicles, small amount of labeling was detected at active zone area colse to the plasma membrane. There was very negligible signal at other compartments of synapses. Given our biochemical fractionation data (Fig. 2–4), it is reasonable to assume that Y188 is detecting both full-length APP and β–CTF localized in CA1 pre-synaptic vesicles.

### The synaptic interactome of the intracellular region of APP revels a potential role of APP in pre-synaptic vesicle exocytosis, endocytosis and recycling

**Figure 8:**
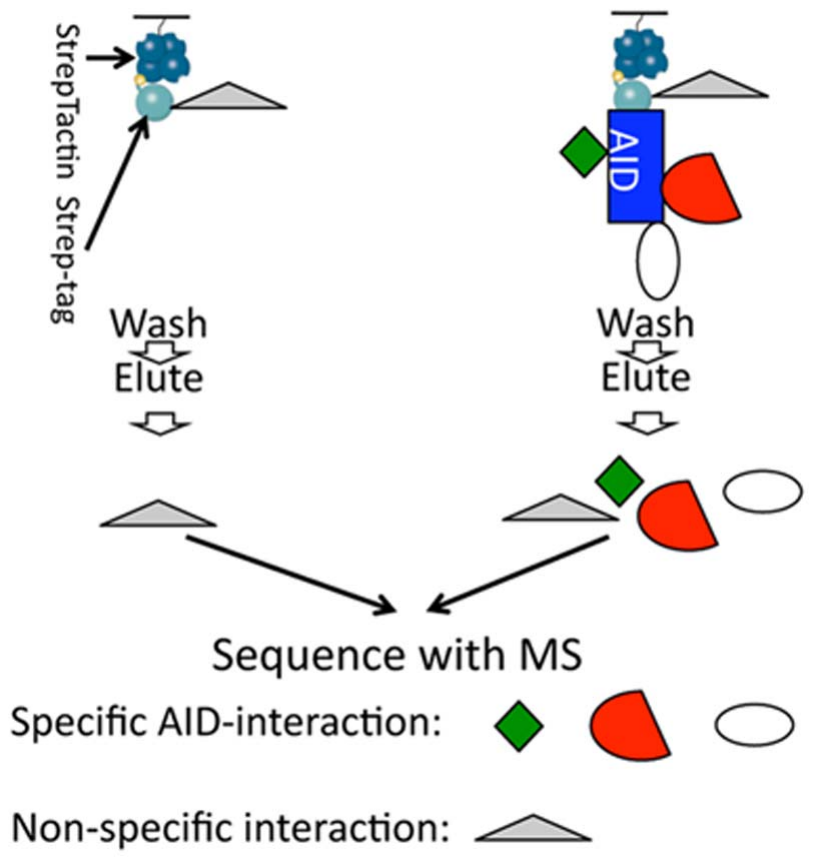
Schematic explanation of the proteomic method used to determine the synaptic interactome of the intracellular domain of APP. St and St-AID peptides were immobilized on StrepTactin resin. Mouse brain fractions were applied on columns; proteins were eluted, digested with trypsin and analyzed by nano LC/MS/MS.

**Figure 9:**
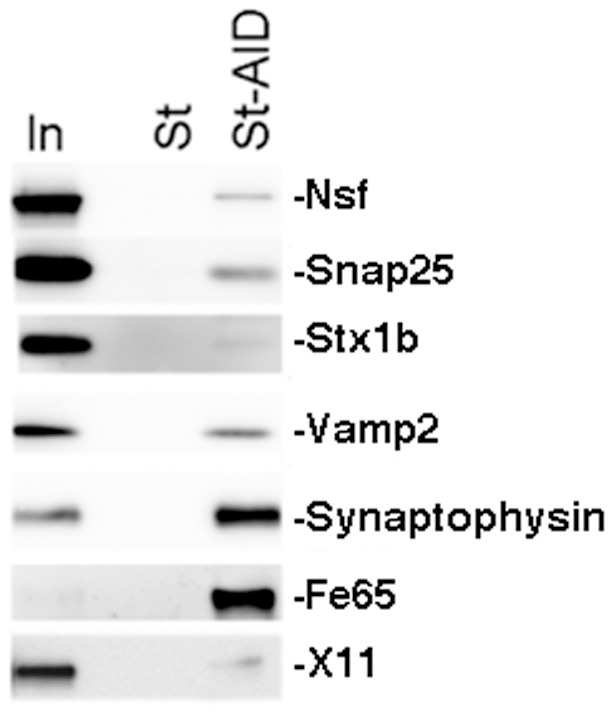
Pre-synaptic proteins that regulate pre-synaptic vesicles endocytosis bind to the intracellular domain of APP. Western blot analysis of pull-downs shows that Nsf, Snap25, Stx1b, Vamp2 and Synaptophysin specifically bind St-AID but not St peptides. The evidence that two previously known APP-interactors, Fe65 and X11, bind St-AID validate the proteomic approach used. In indicates the input.

Based on the structure of APP and the biogenesis of pre-synaptic vesicles, it can be predicted that APP is oriented with the long (∼620 amino-acids) NH_2_-terminus toward the pre-synaptic vesicle's lumen and the short (∼50 amino-acids) intracellular region toward the cytosol of pre-synaptic termini. The evidence that sAPPβ is found in the SV fraction (Fig. 5) is consistent with this prediction. Thus, it is plausible that the NH_2_-terminal domain of APP may have an intra-lumen function while the COOH-terminus may regulate pre-synaptic vesicle's functions *via* interaction with proteins expressed on the outer membrane of synaptic vesicles (i.e. the side exposed to the cytosol), the cytosolic pre-synaptic environment and/or the inner side of the pre-synaptic membrane (active zone). In this context it is worth noting that the short APP intracellular region contains motifs that function as docking domains for cytosolic as well as other membrane-bound proteins [39]–[52]. Although intra- and extra-lumen functions of APP may be both important in pre-synaptic vesicles biology, we decided to explore the potential role of the intracellular region of APP because *in vivo* observations have underlined the key physiological and pathological role of the APP intracellular domain. With this goal in mind, we used a proteomic approach that has been successful in the past [42]. Two synthetic peptides, *i.e.* control Strep-tag peptide (St) and Strep-tag-AID (St-AID), were immobilized on StrepTactin resin. Mouse brain fractions were first passed twice through StrepTactin resin columns to remove proteins that bind the StrepTactin resin. Then, they were applied in parallel on separate columns packed with either StrepTactin-St or StrepTactin-St-AID coated resin. After extensive washings the St and St-AID peptides were eluted, together with proteins specifically bound to them, with desthiobiotin. Eluted proteins were digested with trypsin and identified by nano LC/MS/MS (Fig. 8). Interestingly, this analysis showed that St-AID brings down 18 integral pre-synaptic vesicles proteins (including the vesicular-SNARE Vamp2 and Vamp1, and the Ca^2+^ sensors Synaptotagmin-1 [53] and Synaptotagmin-2), 11 proteins associated with the outer membrane of pre-synaptic vesicles (including Rab proteins, AP-2 subunits, clathrin and complexin) and other pre-synaptic proteins including the target-membrane SNAREs Syntaxin-1b, Syntaxin-1a, Snap25 and Snap47, Munc-18, Nsf and α/β/γ-Snaps (Table 1). To verify the proteomic data we analyzed the pull-down in Western blots for some of these putative interactors. As shown in Fig. 9 Snap25, Syntaxin-1b, Nsf, Vamp2 and synaptophysin were readily detected in St-AID but not in St control pull-downs.

**Table 1:** List of proteins associated with pre-synaptic vesicles and pre-synaptic termini that were included in the APP-Interactome.

Integral vesicle proteins	Associated vesicle proteins	Other pre-synaptic proteins
SCAMP1	AP-2, mu 1 subunit	Synaptosomal-associated protein 25 (Snap25)
SV2	AP-2, mu 2 subunit	Synaptosomal-associated protein 47 (Snap47)
synaptogyrin	AP-2 beta 1 subunit	Syntaxin-binding protein 1 (Stxbp1/Munc-18)
synaptophysin	clathrin heavy chain	Syntaxin-1A (Stx1a)
synaptoporin	complexin	Syntaxin-1B (Stx1b)
synaptotagmin-1	rab1A	γ-soluble NSF attachment protein (SNAPγ)
synaptotagmin-2	rab1B	α-soluble NSF attachment protein (SNAPα)
synaptotagmin-12	rab1C (rab35)	β-soluble NSF attachment protein (SNAPβ)
vATPase V0 subunit a1	rab2	N-ethylmaleimide-sensitive factor (Nsf)
vATPase V0 subunit d1	rab3A	
vATPase V1 subunit A1	rab3C	
vATPase V1 subunit B2, brain isoform		
vATPase V1 subunit C		
vATPase V1 subunit D		
vATPase V1 subunit E		
VAMP I		
VAMP II		
vGLUT1		

Many of these pre-synaptic proteins that constitute the AID-pre-sy-iome play important roles in pre-synaptic vesicles function. Rab proteins, AP-2 subunits and Clathrin are involved in pre-synaptic vesicles endocytosis and recycling [54]–[56]. The vesicular SNARE Vamp2/Vamp1, the target-membrane SNAREs Syntaxin-1b/Syntaxin-1a and Snap25/Snap47, and Munc18 compose the *trans*-SNARE-complex (i.e. a complex composed by proteins on both the pre-synaptic vesicles and active zone membranes) that mediates exocytosis of pre-synaptic vesicles [57]–[59]. Ca^2+^ controls the SNARE and SM fusion machine *via* complexin and the Ca^2+^ sensors synaptotagmins [53], [57]. Following exocytosis, the SNARE complex is found on a single membrane -and is therefore designed as *cis*-SNARE complex. The *cis*-SNARE complex is dissociated into monomers by Nsf and α/β/γ-SNAPs, and vesicles recycle to start another exocytosis round. Overall, the data suggest that APP (and β-CTF) present on synaptic vesicles may play, *via* the interactome of their intracellular domain, a role in synaptic vesicles exocytosis, endocytosis and/or recycling.

## Discussion

Mutations in *APP*, *PSEN1/PSEN2* -which code for the catalytic subunit of the γ-secretase complex- and *BRI2/ITM2B* -which codes for an inhibitor of APP processing by BACE1- cause familial forms of AD and the AD-like Familial Danish Dementia [1], [7]–[12]. Thus, inherited mutations that alter APP processing by either γ- or β-secretases provoke, with 100% penetrance, familial forms of neurodegenative diseases characterized by memory loss.

Additional human genetic data suggest that APP processing is also involved in the most common sporadic forms of AD. In fact, a polymorphism in *APP* that reduces APP processing by BACE1 protects humans from sporadic AD and normal aging-dependent cognitive decline [60]. Moreover, the inhibitor of BACE1 processing of APP BRI2/ITM2B is a “master regulator” of the common pattern of gene expression shared by *ApoE4* carriers, the strongest genetic risk factor for sporadic AD, and sporadic AD patients who do not carry the *ApoE4* allele [61]. Together, the evidence establishs a direct connection in humans between processing of APP by BACE1 and sporadic AD.

Thus, these human genetic data suggest that APP and its processing by β- and γ-secretase is physiologically important for synaptic transmission, synaptic plasticity and memory formation. This hypothesis is supported by *in vitro* data and studies with animal models, which have pointed to a synaptic function for APP and APP processing. Long Term Potentiation (LTP), a form of synaptic plasticity that underlies memory formation, is reduced in *APP* KO mice; furthermore, hippocampal *APP* KO neurons show increased size of the readily releasable synaptic vesicle pool and enhanced amplitudes of evoked AMPA- and NMDA-receptor-mediated responses [62]–[64]. Bace1 cleavage of APP is activated by synaptic activity and, as discussed earlier, generates the β-CTF APP metabolite that exerts a pathological role in mouse models of dementia.

To better understand the synaptic function of APP, we have combined protein chemistry and electron microscopy to study APP, APP-derived metabolites, the APP-cleaving enzymes Bace1 and γ-secretase, in syanpses from mouse brain. Consistent with previous reports [36], our biochemical fractionation data link APP and Bace1 to pre-synaptic vesicles. Moreover, these experiments indicate that Bace1 cleaves APP in pre-synaptic vesicles. The ultrastrural localization of APP in synapses by I-EM revealed that the majority of APP labeling is associated with pre-synaptic vesicles.

As a first step forward in exploring the molecular and biochemical mechanisms underlying the synaptic functions of APP, we have characterized the synaptic interactome of the intracellular region of APP using an unbiased proteomic approach. We focused on this short APP domain because i*n vivo* data have stressed the centrality of this domain to APP's physiological and pathological functions. Consistent with the predominant localization of APP in pre-synaptic termini and more specifically pre-synaptic vesicles, the synaptic interactome of the intracellular region of APP consists mainly of pre-synaptic proteins, either expressed on pre-synaptic vesicles or associated with the active zone. Thus, it is conceivable that the APP and β-CTF molecules present in pre-synaptic vesicles have functional roles. In particular, it is noteworthy the association of the APP intracellular domain with *cis*- and *trans*-SNARE complexes, Rab proteins, AP-2 subunits, complexin and the Ca^2+^ sensors synaptotagmins. These proteins play a central role in regulating exocytosis, endocytosis and recycling of pre-synaptic vesicles [53]–[59]. The AID-pre-sy-iome that we describe in this study is fully consistent with recent work by Norstrom *et al.* and Kohli *et al.* Using transgenic mice over-expressing a tagged APP transgene, these authors have shown that in mouse brain APP can associate with proteins involved in pre-synaptic vesicle cycling/trafficking [65], [66]. Our experiments extend the pre-synaptic APP interactome to additional pre-synaptic proteins and, most importantly, map these interactions to the intracellular domain of APP.

During exoxytosis and disassembly by Nsf and Snaps, SNARE proteins are susceptible to mis-folding. These mis-folding events are controlled and reversed by the classical chaperone complex -containing Cspα, Hsc70 and Sgt- or the non-classical chaperones α/β/γ-synucleins [57], [67]–[71]. These chaperons bind unfolded SNARE proteins and promote their correct folding. Interestingly, none of those chaperons are included in the AID-pre-sy-iome, suggesting that the APP intracellular domain interacts specifically with correctly folded *trans*-SNARE and *cis*-SNARE/Nsf-α/β/γ-Snaps complexes, but not unfolded SNAREs.

In conclusion, the biochemical fractionation and I-EM data, together with the proteomic analysis, suggest that APP and β-CTF expressed on pre-synaptic vesicles could regulate, *via* their intracellular domain, exocytosis, endocytosis and/or recycling of pre-synaptic vesicles. These observations spark many questions. Primarily, does the intracellular region of APP really modulate exocytosis, endocytosis and/or recycling of pre-synaptic vesicles? If so, what is the molecular and biochemical mechanism(s) underlying this function? More experiments will be required to address these important questions, including the biochemical characterization of the protein, or protein complex, that interacts directly with the intracellular region of APP.

Both APP and β-CTF contain the same intracellular region. Therefore, it would be obvious to presume that APP and β-CTF expressed on pre-synaptic vesicles share a common AID-pre-sy-iome and, therefore, similar functions. However, it is possible to envision several scenarios in which processing of APP by Bace1 in pre-synaptic vesicles changes and/or modulates the pre-synaptic role of APP. For example, by cleaving APP into sAPPβ and β-CTF, Bace1 could dissociate the intra-lumen form the extra-lumen functions of APP. Thus, while full-length APP would have both functions, β-CTF would only regulate functions that depend on the AID-pre-sy-iome. In addition, Bace1 processing of APP may result into conformational changes of the intracellular domain of the resulting β-CTF. As a consequence of this conformational change the AID-pre-sy-iome of full-length APP could be different from the AID-pre-sy-iome of β-CTF, which may translate into functional differences between full-length APP and β-CTF. Given the evidence that increased APP processing by Bace1 leads to human dementia and that β-CTF can have a pathogenic role, it will be important to understand whether full-length APP and β-CTF can differentially regulate pre-synaptic vesicles exocytosis, endocytosis and recycling. Thus, studying the function of pre-synaptic APP and β-CTF may help clarifying not only the biochemical mechanisms by which APP exerts its function in synaptic plasticity and memory, but also the molecular mechanisms that lead to dementia.

## Materials and Methods

### Mice and Ethics Statement

Mice were maintained on a C57BL/6 background for several generations (at least 15). Mice were handled according to the Ethical Guidelines for Treatment of Laboratory Animals of Albert Einstein College of Medicine. The procedures were described and approved by the Institutional Animal Care and Use Committee (IACUC) at the Albert Einstein College of Medicine in animal protocol number 20130509.

### Mouse brain preparation

Brains were homogenized (w/v = 10 mg tissue/100 ml buffer) in tissue homogenization buffer (HB: 320 mM Sucrose, 20 mM Tris-base pH 7.4, 1 mM EDTA) supplemented with protease and phosphatase inhibitors (ThermoScientific). Brain homogenates were centrifuged at 800 g for 10 min. Supernatant was collected and centrifuged at 9200 g for 10 min to obtain the pellet (P2) and the supernatant (S2) fractions. The P2 fraction was resuspended in 2 ml of HB 1× and placed on the Percoll gradient. The discontinuous Percoll gradient was composed of 2,5 ml of 23% Percoll, 3 ml of 10% Percoll, 2,5 ml of 3% Percoll, in HB 2× (Sucrose 0.64 M, EDTA 2 mM, Tris Hcl 20 mM ph 7.4). The gradient was centrifuged at 18900 g for 10 min. The material between the 23% and 10% Percoll was transferred in a 50 ml falcon, diluted in 20 ml Krebs buffer (NaCl 140 mM, KCl 5 mM, NaHCO_3_ 5 mM, MgSO_4_ 1.3 mM, Na_2_HPO_4_ 1 mM pH 7.4, Tris/Hepes 10 mM pH 7.4) and centrifuged at 18900 g for 10 min. The pellet, containing synaptosomes (SP) was in some cases resuspended in Krebs buffer (when SP were analyzed) or was resuspended in 1% TritonX100 RIPA buffer, lysed in cold room for 30 min and than centrifuged at 20000 g to obtain the Triton insoluble fraction (pellet) and the triton Soluble fraction (supernatant). The S2 fraction was centrifuged at 55000 g for 1 hr in a TL-100 using the rotor TLA-110. The pellet (p43) was resuspended in HB buffer while the supernatant was centrifuged again for 1 hr at 155000 g. The pellet (SV) was resuspended in HB. The supernatant is designed S74.

### Immunoblot analysis

Samples were separated on 4–20% SDS-PAGE and transferred onto nitrocellulose membranes. The following antibodies were used in Western blots: anti-APP C-terminal AbD (Zymed), anti-APP N-terminal 22C11 (Millipore), anti-sAPPβ (antibodies-online ABIN927102), anti-APPpThr^668^ (Cell Signaling Technology), anti-Bace1 (Cell Signaling Technology), anti-Ps-1 (Cell Signaling Technology), anti-Ps-2 (Cell Signaling Technology), anti-Pen2 (Cell Signaling Technology), anti-Nicastrin (Cell Signaling Technology), anti-Synaptotagmin (Sigma-Aldrich), anti-Synaptophysin (Cell Signaling Technology), anti-Rab3A (Cell Signaling Technology), anti-Synaptobrevin/Vamp2 (Synaptic System), anti-Vdac (Cell Signaling Technology), Rab4 (Cell Signaling Technology), transferrin receptor (Sigma-Aldrich), anti-NmdaR2A (Cell Signaling Technology), anti-NmdaR2B (Cell Signaling Technology), anti-Gapdh (Cell Signaling Technology), anti-Nsf (Cell Signaling Technology), anti-Snap25 (Cell Signaling Technology), anti-Sx1b (Synaptic System). For the experiment reported in Fig. 4, the protein samples were separated on a 16.5% Tris-Tricine SDS-PAGE, to obtain a better separation of APP-CTF species.

### Immuno electron microscopy

Brains were fixed in 4% paraformaldehyde and 0.1% glutaraldehyde in PBS, cryoprotected in 2.3 M sucrose overnight at 4°C. They were transferred to aluminum pins and frozen rapidly in liquid nitrogen. The frozen blocks were cut in liquid nitrogen on a Leica EM FC6 cryomicrotome, 60 nm-thick sections were collected on formvar/carbon coated grids before labeling procedure. For immunolabeling, grids were placed with section-side down on drops of 0.1 M ammonium chloride for 10 min, then blocked in 1% fish skin gelatin in PBS for 20 min. The labeled grids were incubated in a monoclonal antibody against human APP C-terminus Y188 (Abcam) 1∶50 overnight, rinsed in PBS, then incubated with 10 nM protein A gold (Utrecht UMC) for 30 min. Subsequently grids were rinsed in PBS, fixed in 1% glutaraldehyde for 5 min, rinsed and transferred to a UA/methylcellulose drop for 10 min. Samples were examined in a FEI Tecnai Biotwin TEM at 80 kV. Digital Images were acquired using a Morada CCD camera and iTEM (Olympus) imaging software.

### Pull-down assays with St-AID peptides

The APP peptides with an N-terminal strep-tag and have been described previously [42]. The strep-tagged peptides were immobilized on StrepTactin column (IBA, St. Louis, MO), incubated with StrepTactin pre-cleared WT brain homogenates, washed and eluted with desthiobiotin.

### Mass Spectrometry

#### Sample Preparation

The volume of each sample was reduced to 50 µL by vacuum centrifugation, 20 µL of each concentrated sample was processed by SDS-PAGE using a 10% Bis-Tris NuPAGE gel (Invitrogen) with the MES buffer system the gel was run approximately 2 cm. The mobility region was excised into 10 equal sized segments and in-gel digestion was performed on each using a robot (ProGest, DigiLab) with the following protocol: Washed with 25 mM ammonium bicarbonate followed by acetonitrile. Reduced with 10 mM dithiothreitol at 60°C followed by alkylation with 50 mM iodoacetamide at RT. Digested with trypsin (Promega) at 37°C for 4 h. Quenched with formic acid and the supernatant was analyzed directly without further processing.

#### Mass Spectrometry

Each digest was analyzed by nano LC/MS/MS with a Waters NanoAcquity HPLC system interfaced to a ThermoFisher Q Exactive mass spectrometer. Peptides were loaded on to a trapping column and eluted over a 75 µm analytical column at 350 nL/min; both columns were packed with Jupiter Proteo resin (Phenomenex). The mass spectrometer was operated in data-dependent mode, with MS and MS/MS performed in the Orbitrap at 70,000 and 17,500 FWHM resolution, respectively. The fifteen most abundant ions were selected for MS/MS.
